# The role of rare copy number variants in early‐onset depression

**DOI:** 10.1002/jcv2.70128

**Published:** 2026-04-17

**Authors:** Charlotte A. Dennison, Ida Sønderby, Miguel Garcia‐Argibay, Victoria Powell, Amy Shakeshaft, Lucy Riglin, Elliott Rees, Michael O’Donovan, Henrik Larsson, Ole Andreassen, Alexandra Havdahl, Joanna Martin, Anita Thapar

**Affiliations:** ^1^ Wolfson Centre for Young People's Mental Health Cardiff University Cardiff UK; ^2^ Centre for Neuropsychiatric Genetics and Genomics Cardiff University Cardiff UK; ^3^ Department of Medical Genetics Oslo University Hospital and University of Oslo Oslo Norway; ^4^ Centre for Precision Psychiatry Institute of Clinical Medicine University of Oslo Oslo Norway; ^5^ KG Jebsen Centre for Neurodevelopmental Disorders University of Oslo Oslo Norway; ^6^ Developmental EPI Lab Centre for Innovation in Mental Health Faculty of Environmental and Life Sciences University of Southampton Southampton UK; ^7^ School of Medical Sciences Faculty of Medicine and Health Örebro University Örebro Sweden; ^8^ Department of Medical Epidemiology and Biostatistics Karolinska Institutet Stockholm Sweden; ^9^ PsychGen Centre for Genetic Epidemiology and Mental Health Norwegian Institute of Public Health Oslo Norway; ^10^ Research Departement Lovisenberg Diakonale Hospital Oslo Norway; ^11^ Promenta Research Centre Department of Psychology University of Oslo Oslo Norway

**Keywords:** ALSPAC, CATSS, copy number variants, depression, MCS, MoBa

## Abstract

**Background:**

Depression is a highly heterogeneous condition. Depression with an onset in childhood and early adolescence has a worse clinical course, is more heritable, and shows a lower genetic correlation with other depression subtypes, than does later‐onset depression. It is also more strongly associated with neurodevelopmental (ND) comorbidities and genetic liability to attention‐deficit hyperactivity disorder. Thus, we hypothesised that early‐onset depression represents a distinctive ‘neurodevelopmental’ depression subtype associated with an increased burden of rare copy number variants (CNVs) that are enriched in ND conditions. We tested this hypothesis using four population cohorts across the UK, Norway, and Sweden.

**Methods:**

Participants were ascertained from four population cohorts across the UK, Norway, and Sweden. Early‐onset depression was defined as a score >11 on the self‐reported Short Mood and Feelings Questionnaire between ages 10 and 14 years (cases *n* = 5994 vs. controls *n* = 26,388) and, for secondary analyses, using ICD‐10 criteria for major depressive disorder (MDD) with onset ≤14 years (cases *n* = 856 vs. controls *n* = 96,769). Carriers of large, rare (>500 kb, <1% frequency) CNVs and known ND CNVs were identified. Primary analyses tested associations between early‐onset depression and (i) large, rare CNVs, and (ii) ND CNVs. Secondary analyses investigated parent‐reported measures of early‐onset depression.

**Results:**

Meta‐analysis did not identify any robust associations between early‐onset depression (SMFQ‐defined) and large, rare CNVs (OR = 0.92 [95% CI = 0.84–1.02], *p* = 0.12) or ND CNVs (OR = 1.06 [0.85–1.31], *p* = 0.60). No robust associations were observed between early‐onset depression, defined using ICD‐10 MDD criteria, and large rare CNVs (OR = 1.08 [0.86–1.36], *p* = 0.49) or ND CNVs (OR = 0.69 [0.34–1.39], *p* = 0.30).

**Conclusion:**

Our findings did not support the hypothesis that individuals with early‐onset depression show enrichment for large, rare or known ND CNVs.

## Key Points

**What's known?**

- Early‐onset depression (≤14 years) has a worse clinical course and is associated with greater neurodevelopmental (ND) comorbidity than later‐onset depression
- Copy Number Variants (CNVs) are a form of rare genetic variation associated with ND and some psychiatric conditions

**What's new?**

- The authors did not identify any strong evidence to suggest rare or ND CNVs play a role in the aetiology of early‐onset depression

**What's relevant?**

- Early‐onset depression should not be used to guide decisions around CNV testing in child and adolescent psychiatric services
- Future research should explore mechanisms behind the association between early‐onset depression and ND difficulties

## INTRODUCTION

Depression is a common psychiatric condition that is highly heterogeneous in its symptom presentation, age at onset, aetiology, clinical course, and treatment response (Goldberg, 2011). This substantial degree of heterogeneity has led to efforts to stratify depression into more homogenous subtypes (Cai et al., 2020). Several studies have suggested that depression that onsets early (i.e., in childhood) may be phenotypically and genetically different from depression that onsets later in life. Whilst the median age at onset of major depressive disorder (MDD) is 30 years old, 13% of affected individuals experience depression onset by 18 years of age, and 3% by 14 years of age (Solmi et al., 2022). Depression that onsets in childhood or early adolescence is associated with a clinical course that is more chronic and recurrent, a greater likelihood of hospitalisation, and is more strongly associated with ND comorbidities such as autism spectrum diagnosis (ASD) and attention‐deficit hyperactivity disorder (ADHD) than depression that onsets in later adolescence or adulthood (Dennison et al., 2024; Doering et al., 2022; Jaffee et al., 2002; van Os et al., 1997).

Family and twin studies suggest that differences in age at onset of depression index differences in genetic/familial risk. The largest study to date, using Swedish registry data, found that youth‐onset depression (<21 years) showed significantly higher heritability than adult‐onset depression (>25 years) (Nguyen et al., 2023). Whilst genetic correlations between other depression subtypes (e.g., with vs. without psychiatric comorbidity, impairment, suicidality) were substantial (rg = 0.75–0.9), this was not the case for youth‐onset versus adult‐onset depression (rg = 0.33). This finding suggests that youth‐onset depression may represent a partially distinct depression subtype, whilst the other depression subtypes noted above may not reflect substantial differences in genetic liability. Molecular genetic studies offer the additional opportunity of providing biological insights into depression heterogeneity. Consistent evidence of distinct depression subtypes based on common genetic variant profiles (Howard et al., 2020) has not yet been found. However, genome‐wide association studies identify different risk loci for early and late‐onset depression, and early‐onset depression shows significantly stronger genetic correlations with ND conditions than does later‐onset depression (Shorter et al., 2025). Additionally, higher polygenic scores for ADHD, bipolar disorder, and schizophrenia are associated with depression that onsets earlier in adolescence (Musliner et al., 2019; Weavers et al., 2021). These clinical and genetic studies have led to the hypothesis that child/early adolescent‐onset depression may represent a ‘neurodevelopmental’ subtype of depression (Rice et al., 2019).

Rare chromosomal micro‐deletion and micro‐duplication copy number variants (CNVs) are enriched in many ND conditions including intellectual disability/developmental delay, ASD, ADHD, and schizophrenia (Coe et al., 2014; Pinto et al., 2010; Rees et al., 2014; Williams et al., 2010). Evidence for association between known ND CNVs and depression however is inconsistent (Birnbaum et al., 2022; Kendall et al., 2019; Vaez et al., 2024). ND CNVs were associated with diagnosed depression in population‐ascertained biobanks in the UK and the US (Birnbaum et al., 2022; Kendall et al., 2019), and with self‐reported depression in the UK Biobank (Kendall et al., 2019). However, in the Danish patient record‐based iPSYCH sample, ND CNVs were not associated with diagnosed depression (Vaez et al., 2024). Given evidence of a higher ND burden, in terms of clinical phenotypes and common genetic variants, in very early‐onset depression (childhood and early adolescence), we aimed to further test the hypothesis that early‐onset depression represents a ‘neurodevelopmental’ subtype of depression.

In this study we assembled data across four population‐based cohorts from the UK, Norway, and Sweden, to examine whether large, rare CNVs and known ND CNVs are associated with early‐onset depression.

## METHODS

### Participants

Participants were ascertained from four population‐based cohorts: (1) UK Avon Longitudinal Study of Parents and Children (ALSPAC), (2) UK Millennium Cohort Study (MCS), (3) Norwegian Mother, Father, and Child cohort Study (MoBa), and (4) Child and Adolescent Twin Study in Sweden (CATSS).

#### ALSPAC

Pregnant women residing in Avon, UK with an expected delivery date between 1st April 1991 and 31st December 1992 were invited to participate in ALSPAC. A total of 15,658 children were enroled into the study across two waves of recruitment, of whom 14,901 were alive at 1 year of age (Boyd et al., 2013; Fraser et al., 2013; Northstone et al., 2019). The study website contains details of all the data that are available through a fully searchable data dictionary and variable search tool https://www.bristol.ac.uk/alspac/researchers/our‐data/.

#### MCS

MCS recruited children born between 1st September 2000 and 11th January 2002 in sampling areas across the UK. Eligible children were identified through the child benefit register, which captured almost all children living in the UK at the time (Connelly & Platt, 2014). Initial recruitment was completed when children were 9 months old, with a second wave at 3 years resulting in a total sample size of 19,870 children.

#### MoBa

MoBa is a population‐based pregnancy cohort study conducted by the Norwegian Institute of Public Health (NIPH). Participants were recruited from all over Norway from 1999 to 2008. The women consented to participation in 41% of the pregnancies. The cohort includes approximately 114,500 children, 95,200 mothers and 75,200 fathers (Magnus et al., 2016).

#### CATSS

Since July 2004, all twins born in Sweden have been identified through the Swedish Twin Register and are contacted to participate in CATSS when they reach 9 years of age (Anckarsäter et al., 2011). Twins born between July 1992 and June 1995, that is, those aged between 9 and 12 years at the start of recruitment, were contacted at age 12 to participate in the study.

### Ethical information

ALSPAC Ethics and Law Committee and the Local Research Ethics Committees provided ethical approval for the study (project number B3998, approved 22.02.2022). Informed consent for the use of data collected via questionnaires and clinics was obtained from participants following the recommendations of the ALSPAC Ethics and Law Committee at the time. Consent for biological samples has been collected in accordance with the Human Tissue Act (2004). Ethical approval for MCS was obtained from the NHS Research Ethics Committee and parents provided informed consent for their child to participate (project reference GDAC_2021_06_MARTIN, approval 15.03.2022). MoBa is regulated by the Norwegian Health Registry Act. The establishment of MoBa and initial data collection was based on a license from the Norwegian Data Protection Agency and approval from The Regional Committees for Medical and Health Research Ethics. The MoBa cohort is currently regulated by the Norwegian Health Registry Act. The current study was approved by The Regional Committees for Medical and Health Research Ethics (2016/1226/REK sør‐øst C, approved 13.02.2017). Written informed consent was provided by the parents. Ethical approval for CATSS was granted by the Karolinska Institute Ethical Review Board and the Regional Ethical Review Board in Stockholm. Parents provided written consent for their child to participate in CATSS. The CATSS data usage had the following approvals: 02‐289: CATSS9/12 (cohorts born 1992‐2001), 2010/597‐31‐1: CATSS9/12 (cohorts born 2002‐2009), 2016/2135‐31: CATSS9/12 (cohorts born 2010), and 2010/322‐31/2: Registry linkage.

### CNV calling and annotation

DNA was collected from blood samples provided between birth and 7 years in ALSPAC, and samples were genotyped using the Illumina HumanHap 500 quad array. In MCS, DNA samples were collected via saliva at age 14, and genotyped on the Illumina Global Screening Array‐24 v1.0. DNA for MoBa participants was extracted from blood samples taken from the umbilical cord after birth (Paltiel et al., 2014). Samples were genotyped in batches across several arrays: Illumina HumanCoreExome 12v1.1 and 24v1.0, Global Screening Array MD v1.0 and v3.0, and InfiniumOmniExpress‐24 v1.2 (Corfield et al., 2024). Twins recruited to CATSS from 2008 onwards provided saliva samples for DNA extraction; DNA was genotyped using the Illumina Infinium PsychArray‐24 BeadChip.

CNVs were called in each individual cohort using PennCNV, as described previously (Dennison et al., 2025; Martin et al., 2019). CNV quality control was performed separately for each cohort, with the following inclusion filters applied: Log R Ratio (LRR) standard deviation (SD) < 2.5 SD from mean, number of CNVs per individual <100, waviness factor <2.5 SD from mean, number of probes ≥20, confidence score ≥10. We examined large, rare CNVs (<1%) as well as known ND CNVs because both have been implicated as increasing risk for child ND conditions (Martin et al., 2019). For large, rare CNVs, plink v1.07 was used to identify CNVs >500kb with a population frequency <1%. Frequency was determined within each sample separately, consistent with previous literature (Fu et al., 2022; Martin et al., 2019; Pinto et al., 2010). For ND CNVs, 54 CNVs (Coe et al., 2014) were identified using criteria described by Kendall et al. (2017) (Supporting Information S1: Table S1). Most ND CNVs were required to span >50% of the critical region, defined for each CNV in Supporting Information S1: Table S1, except for single gene CNVs where deletions must have covered at least one exon, whilst duplications had to cover the whole gene.

### Depression definition

Our primary outcome was early‐onset depression (onset ≤14 years) defined using the self‐reported Short Mood and Feelings Questionnaire (SMFQ). Participants scoring >11 on the self‐reported SMFQ, the cut‐point validated for depression in this age group (Angold et al., 1995; Thabrew et al., 2018), were defined as having early adolescent‐onset depression. The SMFQ was administered at the following ages: 10, 12, and 13 years in ALSPAC, 14 years in MCS and 14 years in MoBa. Self‐reported SMFQ data were not collected in CATSS. As ALSPAC included multiple timepoints with eligible data, participants meeting the threshold at any time point were defined as having early‐onset depression. We chose 14 years as the maximum age for early‐onset in our study due to evidence that depression prevalence increases sharply after this age (Rice et al., 2019; Solmi et al., 2022), consistency with previous literature (Dennison et al., 2024; Weavers et al., 2021), and data availability across the cohorts.

Our secondary outcome was an episode of depression meeting ICD‐10 criteria of MDD with onset ≤14 years. In ALSPAC, ICD‐10 depression was assessed using the Development and Wellbeing Assessment (Goodman et al., 2000), a structured interview conducted with a parent to ascertain ICD‐10 diagnoses. Participants meeting criteria for MDD at ages 10 or 13 were defined as having early‐onset depression. MoBa and CATSS are linked to the Norwegian and Swedish patient registries, respectively, which report any ICD‐10 diagnoses the person has received in a secondary care setting (e.g., hospital, outpatient clinics, or from contract specialists). Depression in children and adolescents is treated in secondary not primary care in these countries. Individuals receiving a diagnosis of a depressive episode (F32, F33) ≤14 years were defined as having early adolescent‐onset depression, individuals without a diagnosis, or with an MDD diagnosis at an older age, were included as controls. An ICD‐10 diagnosis of depression was not available in MCS.

Given that previous studies have highlighted low agreement between parent and child ratings of depressive symptoms (Baumgartner et al., 2020), and that the predictive validity of parent and child ratings differs across childhood and adolescence (Lewis et al., 2012), we conducted sensitivity checks using parent‐reported SMFQ to define early‐onset depression. Parent‐reported early adolescent‐onset depression was defined as a score >11 on the parent‐reported SMFQ at ages 10, 12, or 13, in ALSPAC, and ages 9 or 12 in CATSS. Parent‐reported data were not available in MCS or MoBa for the full SMFQ.

### Data availability

ALSPAC data are managed by the University of Bristol and are available to bona fide researchers upon application. Researchers can apply for ALSPAC data access on the University of Bristol website: https://www.bristol.ac.uk/alspac/researchers/access/. Phenotypic data for MCS are available to download freely from the UK Data Service https://doi.org/10.5255/UKDA‐Series‐2000031. To access genetic data for MCS, researchers must apply through the Centre for Longitudinal Studies https://cls.ucl.ac.uk/data‐access‐training/genetic‐data‐and‐biological‐samples/, where applications will be subject to ethical approval.

Data from MoBa and the Medical Birth Registry of Norway used in this study are managed by the NIPH, and can be made available to researchers upon approval from the Regional Committees for Medical and Health Research Ethics (REC), compliance with the EU General Data Protection Regulation (GDPR) and approval from the data owners. The consent given by the participants does not allow for storage of data on an individual level in repositories or journals. Researchers who want access to data sets for replication should apply through helsedata.no. Access to data sets requires approval from The Regional Committee for Medical and Health Research Ethics in Norway and an agreement with MoBa.

Due to restrictions under the Swedish Public Access to Information and Secrecy Act, the data are not publicly available. Data access may be granted to researchers upon request and following a secrecy review. Interested researchers should contact Patrik K.E. Magnusson, Department of Medical Epidemiology and Biostatistics, Karolinska Institutet (Email: patrik.magnusson@ki.se).

### Analysis

Primary analyses tested for association between (i) large, rare CNVs and SMFQ‐defined early‐onset depression, and (ii) ND CNVs and SMFQ‐defined early‐onset depression. For ALSPAC and MCS, we performed logistic regressions to test these associations. For MoBa, we performed conditional logistic regression, conditioning on family ID, to account for relatedness. All regression models were run separately for each cohort, then meta‐analysed using a fixed‐effects model, calculated using the function ‘metagen’ in the R package ‘meta’. A fixed‐effects model was used as depression was defined using the same measure, the same informant, and within the same age range across the cohorts. We tested for association between early‐onset depression and standard deviation of the LRR, B Allele Frequency drift, waviness, and genotyping array in each cohort. Where an association with depression was observed (*p* < 0.05), we included the appropriate variable as a covariate in all regression analyses for that cohort.

For the secondary analysis, logistic regressions (ALSPAC) and conditional logistic regressions (MoBa, CATSS) were used as above to test association between large rare CNVs and early‐onset ICD‐10 depression diagnosis, and the association between ND CNVs and early‐onset ICD‐10 depression diagnosis. Results across cohorts were meta‐analysed as with the primary analyses.

Sensitivity analyses were conducted for parent‐reported early‐onset depression using logistic regressions in ALSPAC and CATSS, with results meta‐analysed as above. Sex differences were examined by repeating the primary analyses in males‐only and females‐only subsets of the samples and comparing odds ratios and confidence intervals.

## RESULTS

### Descriptives

A total of 107,930 individuals across all cohorts had genomic data that passed QC (ALSPAC *n* = 8360; MCS *n* = 7827; MoBa *n* = 81,125; CATSS *n* = 10,618). Of those passing QC, 32,382 individuals had self‐reported SMFQ data and 97,625 had early‐onset MDD diagnosis data.

13.5% of ALSPAC participants, 15.9% of MCS participants and 21.2% of MoBa participants experienced self‐reported early‐onset depression, defined using the SMFQ. Rates of early‐onset ICD‐10 depression diagnosis, defined via healthcare records or structured interview, were 1.2% in ALSPAC, 0.8% in MoBa, and 0.9% in CATSS.

9.3% (range 7.5%–10.7%) of individuals across all cohorts carried a large, rare CNV, whilst 1.6% (range 1.4%–2.3%) of individuals carried an ND CNV. The number of individuals carrying CNVs with and without self‐reported early‐onset depression and early‐onset MDD are presented in Table 1.

**TABLE 1:** Numbers of individuals in each cohort with early‐onset depression (self‐reported SMFQ) and early‐onset MDD (clinical diagnosis or structured interview) with and without a large, rare CNV or an ND CNV.

Cohort	Depression assessment	Case‐control status	Large, rare CNV		ND CNV	
			Non‐carrier	Carrier	Non‐carrier	Carrier
ALSPAC	Self‐reported depression	No depression	4802 (91.6%)	441 (8.4%)	5126 (97.8%)	117 (2.2%)
		Depression	747 (91.3%)	71 (8.7%)	796 (97.3%)	22 (2.7%)
	MDD diagnosis	No MDD	5366 (91.3%)	510 (8.7%)	5744 (97.8%)	132 (2.2%)
		MDD	66 (90.4%)	7 (9.6%)	73 (100%)	0 (0%)
MoBa	Self‐reported depression	No depression	13,333 (90.8%)	1353 (9.2%)	14,485 (98.6%)	201 (1.4%)
		Depression	3608 (91.1%)	351 (8.9%)	3904 (98.6%)	55 (1.4%)
	MDD diagnosis	No MDD	72,733 (90.5%)	7650 (9.5%)	79,221 (98.6%)	1162 (1.4%)
		MDD	617 (89.6%)	72 (10.4%)	682 (99.0%)	7 (1.0%)
MCS	Self‐reported depression	No depression	5746 (89.0%)	710 (11.0%)	6322 (97.9%)	137 (2.1%)
		Depression	1109 (91.1%)	108 (8.9%)	1190 (97.8%)	27 (2.2%)
CATSS	MDD diagnosis	No MDD	9718 (92.5%)	792 (7.5%)	10,338 (98.4%)	172 (1.6%)
		MDD	88 (93.6%)	6 (6.4%)	>89 (>94.7%)	<5 (<5.3%)

*Note*: Cell counts <5 are not specified to maintain anonymity.

Abbreviations: ALSPAC, Avon Longitudinal Study of Parents and Children; CATSS, Child and Adolescent Twin Study in Sweden; CNV, copy number variant; MCS, UK Millennium Cohort Study; MDD, major depressive disorder; MoBa, Norwegian Mother, Father, and Child cohort Study; ND, neurodevelopmental; SMFQ, self‐reported Short Mood and Feelings Questionnaire.

### Early‐onset depression – self‐reported questionnaire

**FIGURE 1:**
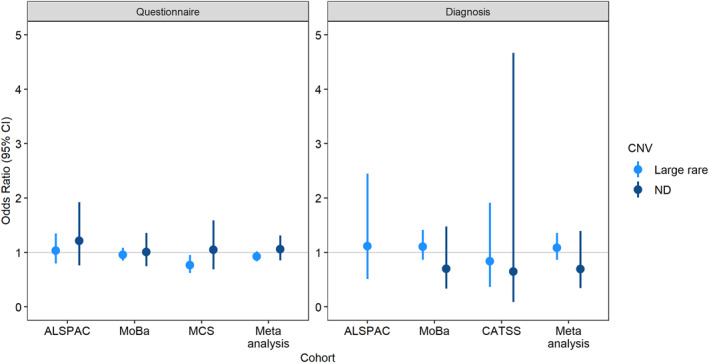
Results of analysis testing associations between early‐onset depression and large rare CNVs (light blue) and ND CNVs (dark blue). Results are presented with a definition of depression based on either self‐rated questionnaire (left panel) or as ICD‐10 diagnosis (right panel). Points display Odds Ratio and bars refer to 95% confidence intervals. CNVs, copy number variants; ND, neurodevelopmental.

Large, rare CNV carrier status was not associated with self‐reported early‐onset depression, defined using the SMFQ, in the meta‐analysis (OR = 0.92 [95% CI = 0.84–1.02], *p* = 0.12) or in any individual cohort, with the exception of MCS, where carrying a large rare CNV was associated with reduced likelihood of early‐onset depression (Table 2, Figure 1).

**TABLE 2:** Association between CNVs and (i) self‐reported early‐onset depression, defined via the SMFQ, and (ii) early‐onset MDD diagnosis.

Analysis	Cohort	Large CNV				ND CNV			
		OR	Lower CI	Upper CI	*p*‐value	OR	Lower CI	Upper CI	*p*‐value
SMFQ self‐reported depression	ALSPAC	1.03	0.80	1.35	0.80	1.21	0.76	1.92	0.42
	MoBa	0.96	0.85	1.08	0.50	1.01	0.75	1.36	0.97
	MCS	0.77	0.62	0.95	0.01	1.05	0.69	1.59	0.83
	Meta‐analysis	0.92	0.84	1.02	0.12	1.06	0.85	1.31	0.60
MDD diagnosis	ALSPAC	1.12	0.51	2.45	0.78	NA – insufficient cell count			
	MoBa	1.10	0.86	1.41	0.43	0.70	0.33	1.48	0.35
	CATSS	0.84	0.37	1.91	0.67	0.65	0.09	4.67	0.67
	Meta‐analysis	1.08	0.86	1.36	0.49	0.69	0.34	1.39	0.30

Abbreviations: ALSPAC, Avon Longitudinal Study of Parents and Children; CATSS, Child and Adolescent Twin Study in Sweden; CNV, copy number variant; MCS, UK Millennium Cohort Study; MDD, major depressive disorder; MoBa, Norwegian Mother, Father, and Child cohort Study; ND, neurodevelopmental; SMFQ, self‐reported Short Mood and Feelings Questionnaire.

ND CNVs were not associated with depression in the meta‐analysis (OR = 1.06 [0.85–1.31], *p* = 0.60), or in any individual cohort (Table 2, Figure 1).

### Early‐onset MDD diagnosis

Large rare CNVs were not associated with early‐onset depression meeting ICD‐10 criteria for MDD in any cohort or the meta‐analysis (OR = 1.08 [0.86–1.36], *p* = 0.49). No association was observed between ND CNVs and early‐onset depression defined by ICD‐10 MDD criteria in the meta‐analysis (OR = 0.69 [0.34–1.39], *p* = 0.30) or any individual cohort (Table 2, Figure 1).

### Sensitivity analyses – parent‐rated, SMFQ‐defined depression

**FIGURE 2:**
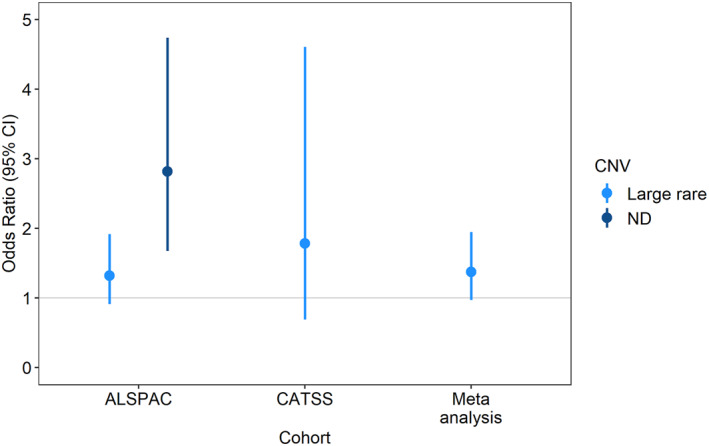
Results of analysis testing associations between parent‐reported, questionnaire‐defined early‐onset depression and large rare (light blue) or (ND, dark blue) CNVs. Points display Odds Ratio and bars refer to 95% confidence intervals. ND, neurodevelopmental; CNVs, copy number variants.

No evidence of association was observed between parent‐reported early‐onset depression and large, rare CNVs in ALSPAC or CATSS, or when the two cohorts were meta‐analysed (Figure 2 and Supporting Information S1: Table S2).

Parent‐reported early‐onset depression was associated with carrying an ND CNV in ALSPAC, but there were insufficient data to test this association in CATSS thus a meta‐analysis could not be performed (Figure 2 and Supporting Information S1: Table S2).

### Sex differences

Full results of the analyses examining sex differences are in Supporting Information S1: Tables S3 and S4 and Figure S1. There was evidence that females carrying a large rare CNV had a reduced likelihood of self‐reported early‐onset depression, which was not observed in males although the confidence intervals for males and females overlapped (Supporting Information S1: Table S3 and Figure S1). No other differences were observed between males and females.

## DISCUSSION

Across four population cohorts totalling 107,930 children and adolescents, we did not find strong evidence to support the hypothesis that rare CNVs are associated with early‐onset depression ≤14 years. No associations were observed between large rare or ND CNVs and depression whether defined by self‐reported SMFQ, or as an episode of depression meeting criteria for an ICD‐10 MDD diagnosis. Our findings suggest that rare CNVs do not play a substantial role in the aetiology of early adolescent‐onset depression.

There is some evidence to suggest that ND CNVs may be associated with depression in adulthood, although findings are inconsistent (Birnbaum et al., 2022; Kendall et al., 2019; Vaez et al., 2024). Nevertheless, CNVs have been consistently associated with an elevated likelihood of ND conditions, including intellectual disability, ASD, and ADHD, that themselves are associated with earlier‐onset depression (Dennison et al., 2024; Doering et al., 2022). There are several reasons that may explain why we did not find evidence of association between ND CNVs and early‐onset depression.

First, the association between early‐onset depression and ND conditions may be explained by shared common genetic liability and/or other forms of rare genetic variation, rather than by rare copy number variants. Developmental trajectories of self‐reported depression symptoms across childhood, adolescence, and adulthood suggest that earlier onset, persistent depression is associated with elevated ADHD polygenic scores (Weavers et al., 2021). Mendelian randomisation studies also suggest a causal relationship between common genetic variants for ADHD and risk of depression (Riglin et al., 2021). This could suggest that the ND component to early‐onset depression is mainly driven by common genetic variation. However, other forms of rare genetic variation may also be important. Studies of single nucleotide variants (SNVs) highlight overlap between the rare coding variants associated with depression and those associated with ND conditions (Cheng et al., 2022; Tian et al., 2024). The contribution of SNVs to youth‐onset depression remains unexplored.

Another possibility is that the higher heritability estimates and worse outcomes associated with early‐onset compared to later‐onset depression may be explained by early environmental adversities that are correlated with genetic risk (i.e., gene‐environment correlation). For instance, adverse childhood experiences, which are increased for children with ND conditions (McDonnell et al., 2019), show correlation with genetic liability (Baldwin et al., 2023; Warrier et al., 2021), and are associated with depression risk (Hughes et al., 2017). Additionally, it is possible that depression may be missed or under‐reported when the individual has complex needs or other urgent health concerns, as is common among CNV carriers who can experience a range of ND and physical health impacts (Chawner et al., 2021). Evidence also suggests that questionnaires assessing depressive symptoms, which are typically validated in neurotypical populations, may not be equally appropriate or valid for neurodivergent individuals (Cassidy et al., 2018), who are more likely to carry a CNV.

Our study relied primarily on self‐reported depression data. Research typically suggests combining both parent and self‐reports for most reliable assessment of youth depression (Cohen et al., 2019). However, in longitudinal cohorts it is rare to have both parent and self‐reports of the same measure assessed at the same time. The age range of participants included in our study covers a period where clinicians and healthcare professionals may move from parent‐report to self‐report (Youngstrom et al., 2011), and it is possible that the point at which self‐report becomes a more reliable informant differs for different individuals and especially for neurodivergent individuals. In particular, adolescents with ADHD have been found to under‐report depressive symptoms compared to parent reports, whilst neurotypical adolescents were found to over‐report depressive symptoms compared to parent reports (Fraser et al., 2018). Indeed, we found evidence in the ALSPAC cohort to suggest that ND CNVs may be associated with parent‐reported depression. However, we were unable to investigate this in the other samples due to lack of data availability. It is important for future studies to consider the impact of informant and measurement during the study design, and particularly how differences across ages and cohorts may affect the findings.

A strength of our study was the consistent measurement of depression across multiple cohorts—that is, the same informant and the same questionnaire within the same age range, which is often difficult to achieve across population cohorts. Despite the large overall sample size, small individual numbers in specific cohorts prohibited some analyses, such as MDD diagnosis and ND CNVs in ALSPAC. Rare CNVs are uncommon by definition and depression onsets at or before 14 years is also rare. Thus, we cannot rule out the possibility that our null finding is a type I error explained by insufficient statistical power. However, power analysis indicated that we had 80% power to detect an odds ratio of 1.27 or larger for the association between ND CNVs and SMFQ‐defined early‐onset depression, and an odds ratio of 1.11 or higher for the association between large rare CNVs and SMFQ‐defined depression. Whilst effect sizes smaller than this could be statistically significant in a larger sample, they would not be meaningful enough to justify recommending routine genetic testing. Due to the rare nature of ND CNVs, we were unable to measure associations with specific types of CNVs and instead created a pooled measure of CNVs known to be associated with ND conditions (Coe et al., 2014). It is possible that some types of CNVs that we were unable to analyse (e.g., CNVs in specific loci, rarer CNVs), may be associated with early‐onset depression, and could explain the associations between early‐onset depression and ND phenotypes.

Overall, our findings suggest that rare CNVs do not substantially increase the likelihood of early‐onset depression, when assessed via self‐report or ICD‐10 diagnosis. Our findings are inconsistent with evidence of enrichment of ND phenotypes in early‐onset depression (Dennison et al., 2024; Doering et al., 2022), but are consistent with some, although not all, evidence from CNV studies of depression in adult populations (Vaez et al., 2024).

## AUTHOR CONTRIBUTIONS

**Charlotte A. Dennison**: Conceptualisation; formal analysis; investigation; methodology; visualisation; writing—original draft. **Ida Sønderby**: Formal analysis; investigation; writing—review and editing. **Miguel Garcia‐Argibay**: Formal analysis; investigation; writing—review and editing. **Victoria Powell**: Investigation; methodology; writing—review and editing. **Amy Shakeshaft**: methodology; writing—review and editing. **Lucy Riglin**: Methodology; writing—review and editing. **Elliott Rees**: Methodology; writing—review and editing. **Michael O'Donovan**: Methodology; writing—review and editing. **Henrik Larsson**: Resources; supervision; writing—review and editing. **Ole Andreassen**: Resources; supervision; writing—review and editing. **Alexandra Havdahl**: Conceptualisation; resources; supervision; writing—review and editing. **Joanna Martin**: Conceptualisation; methodology; supervision; writing—review and editing. **Anita Thapar**: Conceptualisation; funding acquisition; supervision; writing—original draft.

## CONFLICT OF INTEREST STATEMENT

H.L. reports receiving grants from Shire Pharmaceuticals and Takeda; personal fees from and serving as a speaker for Medice, Shire/Takeda Pharmaceuticals and Evolan Pharma AB; all outside the submitted work. H.L. is editor‐in‐chief of JCPP Advances. E.R. and M.O.D. reported receiving grants from Akrivia Health outside the submitted work. M.O.D. reported receiving grants from Takeda Pharmaceutical Company Ltd outside the submitted work. Takeda and Akrivia played no part in the conception, design, implementation, or interpretation of this study. The remaining authors have declared that they have no competing or potential conflicts of interest.

## ETHICAL CONSIDERATION

ALSPAC Ethics and Law Committee and the Local Research Ethics Committees provided ethical approval for the study (project number B3998, approved 22.02.2022). Informed consent for the use of data collected via questionnaires and clinics was obtained from participants following the recommendations of the ALSPAC Ethics and Law Committee at the time. Consent for biological samples has been collected in accordance with the Human Tissue Act (2004). Ethical approval for MCS was obtained from the NHS Research Ethics Committee and parents provided informed consent for their child to participate (project reference GDAC_2021_06_MARTIN, approval 15.03.2022). MoBa is regulated by the Norwegian Health Registry Act. The establishment of MoBa and initial data collection was based on a license from the Norwegian Data Protection Agency and approval from The Regional Committees for Medical and Health Research Ethics. The MoBa cohort is currently regulated by the Norwegian Health Registry Act. The current study was approved by The Regional Committees for Medical and Health Research Ethics (2016/1226/REK sør‐øst C, approved 13.02.2017). Written informed consent was provided by the parents. Ethical approval for CATSS was granted by the Karolinska Institute Ethical Review Board and the Regional Ethical Review Board in Stockholm. Parents provided written consent for their child to participate in CATSS. The CATSS data usage had the following approvals: 02‐289: CATSS9/12 (cohorts born 1992‐2001), 2010/597‐31‐1: CATSS9/12 (cohorts born 2002‐2009), 2016/2135‐31: CATSS9/12 (cohorts born 2010), and 2010/322‐31/2: Registry linkage.

## Supporting information

Supporting Information S1

## Data Availability

ALSPAC data are managed by the University of Bristol and are available to bona fide researchers upon application. Researchers can apply for ALSPAC data access on the University of Bristol website: https://www.bristol.ac.uk/alspac/researchers/access/. Phenotypic data for MCS are available to download freely from the UK Data Service https://doi.org/10.5255/UKDA‐Series‐2000031. To access genetic data for MCS, researchers must apply through the Centre for Longitudinal Studies https://cls.ucl.ac.uk/data‐access‐training/genetic‐data‐and‐biological‐samples/, where applications will be subject to ethical approval. Data from MoBa and the Medical Birth Registry of Norway used in this study are managed by the Norwegian Institute of Public Health, and can be made available to researchers upon approval from the Regional Committees for Medical and Health Research Ethics (REC), compliance with the EU General Data Protection Regulation (GDPR) and approval from the data owners. The consent given by the participants does not allow for storage of data on an individual level in repositories or journals. Researchers who want access to data sets for replication should apply through helsedata.no. Access to data sets requires approval from The Regional Committee for Medical and Health Research Ethics in Norway and an agreement with MoBa. Due to restrictions under the Swedish Public Access to Information and Secrecy Act, the data are not publicly available. Data access may be granted to researchers upon request and following a secrecy review. Interested researchers should contact Patrik K.E. Magnusson, Department of Medical Epidemiology and Biostatistics, Karolinska Institutet (Email: patrik.magnusson@ki.se).
